# Prevalence and associated factors of medication non-adherence in hematological-oncological patients in their home situation

**DOI:** 10.1186/s12885-017-3735-1

**Published:** 2017-11-09

**Authors:** Linda Bouwman, Corien M. Eeltink, Otto Visser, Jeroen J. W. M. Janssen, Jolanda M. Maaskant

**Affiliations:** 1 Department of Hematology, VU University Medical Center, Amsterdam, the Netherlands; 2 Emma Children’s Hospital, Academic Medical Center, Amsterdam, the Netherlands; 3 Department of Clinical Epidemiology, Biostatistics and Bioinformatics, Medical Faculty, Academic Medical Center and University of Amsterdam, Amsterdam, the Netherlands; 4 Cancer Center Amsterdam, VU University Medical Center, De Boelelaan 1117, 1081 HV Amsterdam, The Netherlands

**Keywords:** Non-adherence, Associated factors, Hematological-oncological patients

## Abstract

**Background:**

Medication non-adherence is associated with poor health outcomes and increased health care costs. Depending on definitions, reported non-adherence rates in cancer patients ranges between 16 and 100%, which illustrates a serious problem. In malignancy, non-adherence reduces chances of achievement of treatment response and may thereby lead to progression or even relapse. Except for Chronic Myeloid Leukemia (CML), the extent of non-adherence has not been investigated in hematological-oncological patients in an outpatient setting. In order to explore ways to optimize cancer treatment results, this study aimed to assess the prevalence of self-administered medication non-adherence and to identify potential associated factors in hematological-oncological patients in their home situation.

**Methods:**

This is an exploratory cross-sectional study, carried out at the outpatient clinic of the Department of Hematology at the VU University medical center, Amsterdam, the Netherlands between February and April 2014. Hematological-oncological outpatients were sent questionnaires retrieving information on patient characteristics, medication adherence, beliefs about medication, anxiety, depression, coping, and quality of life. We performed uni- and multivariable analysis to identify predictors for medication non-adherence.

**Results:**

In total, 472 participants were approached of which 259 (55%) completed the questionnaire and met eligibility criteria. Prevalence of adherence in this group (140 male; 54,1%; median age 60 (18–91)) was 50%. In univariate analysis, (lower) age, (higher) education level, living alone, working, perception of receiving insufficient social support, use of bisphosphonates, depression, helplessness (ICQ), global health, role function, emotional function, cognitive function, social functioning, fatigue, dyspnea, diarrhea were found to be significantly related (p = <0.20) to medication non-adherence. In multivariable analysis, younger age, (higher) education level and fatigue remained significantly related (p = <0.10) to medication non-adherence.

**Conclusions:**

This cross-sectional study shows that 50% of the participants were non-adherent. Lower age, living alone and perception of insufficient social support were associated factors of non-adherence in hematological-oncological adult patients in their home-situation.

## Background

Non-adherence, defined as ‘a deviation from the prescribed medication regimen sufficient to adversely influence the regimen’s intended effect’ [1], is associated with poor health outcomes [2] and increased healthcare costs [3, 4]. According to the World Health Organization (WHO) approximately 50% of chronically ill patients who undergo long-term treatment are non-adherent to their medication [5]. A more recent systematic review about patient adherence to oral anti-cancer drugs showed that non-adherence in cancer patients is a significant problem [6]. In several studies, mainly on patients with breast cancer and malignant hematological diseases, depending on definitions and methodology, adherence ranged from between 16 and 100%. [6] Another systematic review about adherence in patients with hematological malignancies reports adherence rates between 20 and 53% in patients with chronic myeloid leukemia (CML) and non-adherence rates of 6–35% in patients with acute lymphoid leukemia (ALL) [7].

Patients treated for malignant hematological diseases, such as acute or chronic leukemias and aggressive lymphomas, often need treatment that involves chemotherapy, immunosuppressive treatment and additional supportive medication to prevent patients from complications like deep venous thrombosis, osteoporosis and infections. Many patients often need multiple oral or topical drugs, self-administered at home, for long periods of time in complex schedules, which, in addition to often experienced side effects, like nausea, diarrhea and fatigue may result in reduced medication adherence. Moreover, socio-economic factors are found to be associated to medication non-adherence [8–10]. Ultimately, depending on the nature of the medication, this may lead to serious complications like infections, graft-versus-host-disease and progression or relapse of the underlying malignancy [11, 12].

As oral anti-cancer drugs are typically taken self-administered in the home setting, adherence is a major issue especially in outpatients. Thus, as shown by Marin et al. (2010) patients taking ≤90% of prescribed tablets of imatinib for chronic myeloid leukemia had clearly inferior major molecular response rates compared to adherent patients. In addition, optimal drug adherence was associated with positive health outcomes [13]. In times of a rapidly growing availability of oral cancer drugs, non-adherence urgently needs to be addressed. [14, 15] Medication non-adherence has been studied in several groups of patients with hematological malignancies, mostly CML and ALL [7–9, 16, 17], however thorough investigations in a population of patients with a variety of hematological malignancies in their home situation is still lacking. This is necessary, because self-administration of oral medications is required for a growing number of cancer treatments, also in case of immunosuppressing drugs and infection prophylaxis. Therefore we set out to assess the extent of non-adherence and to identify potential associated factors in a population of patients with a variety of hematological malignancies in their home situation.

## Methods

### Setting

This exploratory, cross-sectional study in ambulant hematological-oncological patients was conducted at the outpatient clinic of the Department of Hematology at the VU University medical center, Amsterdam. This is a tertiary university hospital which provides care to patients from all over the Netherlands. Patients are treated for a complete range of hematological malignancies. This setting was chosen, because outpatient clinic patients do self-administer their medication in the home setting, while patients admitted to the clinical ward get medication distributed by nurses.

### Participants

Participants with an appointment at the Hematology outpatient clinic in February, March or April 2014 were approached for inclusion in the study. Inclusion criteria were: (1) Treatment for a hematological malignancy at any stage of their disease (2) Use of medication for treating side effects or complications of their treatment for a hematological malignancy (3) At least one prescription medication to be used daily in the home setting (oral, subcutaneous, but for example also eye-drops or ointments used for local treatment of graft-versus-host-disease (4) Age > 18 years and (5) Dutch speaking and writing.

Inclusion criteria were chosen to understand the problem of non-adherence in all adult patients with a hematological malignancy visiting the outpatient clinic. Also patients who deal with side effect or complications from their disease or treatment.

The study was approved by the Ethics Committee of the VU University Medical Center. The study was conducted according to the Declaration of Helsinki, ICH GCP Guidelines, the EU directive for Good Clinical Practice (2001/20/EG).

### Data collection

Data were obtained from questionnaires and patients’ medical files (socio-economic factors and disease). The questionnaires were sent to patients by regular mail a week before their appointment at the outpatient clinic. Patients were asked for informed consent, to complete the questionnaires at their homes and bring them to their next appointment at the outpatient clinic.

### Instruments

Various validated questionnaires, available in Dutch, were used in this study. The Medication Adherence Rating Scale 5 item version (MARS-5) [18, 19], was used to measure the prevalence of non-adherence, because it was the only validated questionnaire in Dutch that measures adherence available. The Beliefs about Medication Questionnaire (BMQ) [20, 21], the Hospital Anxiety and Depression Subscale (HADS) [22–24], the Illness Cognitions Questionnaire (ICQ) [25, 26] and the European Organization for Research and Treatment of Cancer, Quality of Life Questionnaire-C 30 version 3.0 (EORTC QLQ-C30) [27, 28] were used to determine potential correlative factors to predict non-adherence. In addition, we collected information on socio-economic characteristics, disease and addiction, that we considered to be potential associated factors for non-adherence.

#### MARS-5

This questionnaire measures patients’ adherence to medication. Each item can be scored from 1 to 5 (1 = always, 5 = never) resulting in a minimum sum score of 5 and a maximum sum score of 25. The lower the score, the less adherent patients are [18, 19].

The MARS-5 questionnaire is one of many validated questionnaires to measure non-adherence, it was used in this study because it was the only questionnaire available in Dutch. It is not validated in the population of hematology patients.

The MARS-5 has no cut-off value to define adherence. We defined non-adherence as “a deviation from the prescribed medication regimen sufficient to adversely influence the regimen’s intended effect” [1]. In this study, a patient was considered non-adherent when he scored less than the maximum score of 25.

#### BMQ

This questionnaire measures patients’ beliefs about the necessity of their prescribed medication and their concerns about potential consequences of taking the prescribed medication. The scale contains 10 items, which can be scored on a 5-point Likert-scale (1 = strongly disagree, 5 = strongly agree). The higher participants score on the necessity items, the stronger they believe that their prescribed medication is necessary. The higher participants score on the concerns items, the more concerned they are about taking the prescribed medication [20, 21].

#### HADS

This scale measures depression and anxiety in medically ill patients. The HADS is divided into the subscales anxiety and depression, each containing 7 items with sum scores between 0 and 21. A score of 8 or more indicates that a participant might be either anxious or depressed. A score under 8 is considered normal [22–24].

#### ICQ

This is a generic questionnaire that measures illness beliefs in chronically ill patients. The questionnaire consists of 18 items and each item is scored from 1 to 4 (1 = not at all, 4 = completely). The questionnaire contains 3 subscales: helplessness, acceptance, and perceived benefits, each containing 6 items resulting in sum scores from 6 to 24. For each item, higher scores indicate either higher feeling of helplessness, higher acceptance of the underlying illness or higher perceived benefits from being ill [25–27].

#### EORTC QLQ-C30

This questionnaire measures quality of life in cancer patients. It is a 30-item questionnaire including five functional scales (physical, role, cognitive, emotional and social), three symptom scales (fatigue, pain, and nausea and vomiting), a Quality of Life scale, scores for symptoms that often occur in cancer patients (dyspnea, loss of appetite, insomnia, constipation and diarrhea) and for financial problems as a result of the disease. The results on the separate items are converted into scores ranging from 0 to 100. Higher scores indicate a higher quality of life [28, 29].

### Data entry

Quality of data entry was assessed by random sampling of data entries by a second independent person. In total 1.1% errors were found. We corrected the errors after checking the primary data sources.

### Statistical analysis

Descriptive statistics were used to describe the characteristics of the participants, as well as the prevalence of medication non-adherence. We report frequencies and proportions, means and standard deviations, or medians and interquartile ranges when appropriate. Univariable logistic regression was performed to select factors associated with medication adherence. Possible associated factors in the univariate analysis were selected for multivariable regression analysis if associated with adherence (i.e. *p* < 0.20). Living situation was dichotomized into living alone or not alone and work status was dichotomized into working or not working. Continues data was not dichotomized. We investigated potential interaction terms between all items found significant in the multivariable regression analysis. In the multivariable regression model, we considered *P* values <0.10 to be significant. We used the backward selection method in which non-significant items were removed from the model until only significant items were left. Results from the univariate and multivariable regression analysis are expressed as regression coefficients, 95% confidence intervals and *p* values.

Statistical analyses were performed using SPSS (version 20.0. IBM, Armonk, NY, USA).

## Results

### Participants

In total, 472 patients with a hematological malignancy (mostly acute leukemia, chronic leukemia, (non)Hodgkin and multiple myeloma) were included in the study and 280 questionnaires were returned (59.3% response rate). Twenty-one participants were retrospectively excluded, because they did not use prescription medication. Thus, overall, 259 (55%) participants were included in the analysis. Table 1 shows participants’ demographics.

**Table 1:** Demographic and clinical characteristics

Variable	Sample(*n* = 259)	%
Age (median)	60	50–67 (IQR)
Male gender	140	54.1
Education level		
Primary school	8	3.1
Secondary education	74	28.6
Secondary vocational	68	26.3
Bachelor	75	29
Master	25	9.7
Living alone	50	19.3
Living with family/roommates	209	80.7
Work situation		
Unemployed	55	21.2
Employed	70	27
Receive sickness benefit	51	19.7
Retired	81	31.3
Diagnosis		
Acute leukemia	69	26.6
Chronic leukemia*	57	22
(Non)hodgkin*	39	15.1
Multiple myeloma*	73	28.2
Others	21	8.1
Smoking	15	5.8
Alcohol consumption (daily)	56	21.6
Medication		
Anti-cancer medication	101	40.9
Growth factor	16	6.5
Bisphosphonates	51	20.6
Anticoagulants	45	18.2
Antibiotics	138	55.9
Corticosteroids	86	34.8
Immunosuppressants	46	18.6
HADS		
Anxiety >8	55	22.3
Depression >8	52	21,1
ICQ		
Helplessness (median)	12	9–16 (IQR)
Acceptance (median)	17	14–20 (IQR)
Disease benefits (median)	16	12–19 (IQR)
EORTC-QLQ30		
Global health (median)	66.7	58.3–83.3(IQR)
Physical function (median)	80	60–93.3(IQR)
Role function (median)	66.6	33.3–100 (IQR)
Emotional function (median)	83.3	66.7–100 (IQR)
Cognitive function (median)	83.3	36.7–100 (IQR)
Social function (median)	83.3	66.7–100 (IQR)
Fatigue (median)	33.3	22.2–55.6(IQR)
Nausea (median)	0	0–16.7 (IQR)
Pain (median)	16.7	0–33.3 (IQR)
Dyspnea (median)	33.3	0–33.3 (IQR)
Insomnia (median)	33.3	0–33.3 (IQR)
Loss of appetite (median)	0	0–33.3 (IQR)
Constipation (median)	0	0–8.33 (IQR)
Diarrhea (median)	0	0–0 (IQR)
Financial problems (median)	0	0–33.3 (IQR)
BMQ		
Necessity (median)	19	16–23 (IQR)
Concerns (median)	16	13–20 (IQR)

### Prevalence of adherence

Full adherence to their drug regimen (score 25) was reported by 50% of patients (50%). The results on the MARS-5 score varied from 9 to 25. The distribution of non-adherence scores is presented in Table 2.

**Table 2:** Distribution and frequency of MARS scores

MARS-5 score	Frequencies	%
25	130	50,2
24	72	27.8
23	31	12
22	7	2.7
21	3	1.2
20	5	1.9
19	4	1.5
18	3	1.2
15	1	0.4
10	2	0.8
9	1	0.4

Scores on the Medication Adherence Rating Scale 5-item (total score ranges from 5 to 25)

### Univariate analysis

Significant relations were found between adherence and (lower) age (*p* = 0.002), (higher) education level (*p* = 0.062), living alone (*p* = 0.164), working (*p* = 0.197), perception of receiving insufficient social support (*p* = 0.073), use of bisphosphonates (*p* = 0.132), depression (*p* = 0.099), helplessness (ICQ) (*p* = 0.175), global health (*p* = 0.167), role function (*p* = 0.106), emotional function (*p* = 0.114), cognitive function (*p* = 0.028), social function (*p* = 0.027), fatigue (*p* = 0.032), dyspnea (*p* = 0.196), diarrhea (*p* = 0.067). Table 3 presents all the variables included in the univariate analysis.

**Table 3:** Univariable analysis

Variable	*B*	*P* value	95 % CI
Age*	−0.031	0.002	0.950 to 0.989
Sex	0.046	0.857	0.635–1.726
Education level*	0.314	0.062	0.984 to 1.903
Living alone*	−0.461	0.164	0.330 to 1.207
Working*	0.405	0.197	0.811 to 2.772
Acute leukemia	21.002	1	
Chronic leukemia	−0.201	0.695	0.3 to 2.234
(Non)hodgkin	−0.622	0.241	0.190 to 1.517
Multiple myeloma	0.136	0.809	0.380 to 3.449
Others	−0.229	0.653	0.294 to 2.154
Smoking	0.521	0.373	0.535 to 5.3
Alcohol consumption (daily)	0.126	0.683	0.62 to 2.075
Experiencing social support*	1.074	0.073	0.905 to 9.466
Disease education	−0.14	0.746	0.373 to 2.024
Sufficient disease education	−0.461	0.43	0.2 to 1.985
Medication			
Anti-cancer medication	−0.194	0.455	0.496 to 1.370
Growth factor	0.026	0.96	0.373 to 2.827
Bisphosphonates*	0.479	0.132	0.865 to 3.015
Anticoagulants	−0.318	0.246	0.425 to 1.245
Antibiotics	0.253	0.326	0.778 to 2.13
Corticosteroids	0.037	0.889	0.615 to 1.752
Immunosuppressants	0.352	0.285	0.746 to 2.711
Number of medication	0.015	0.563	0.965 to 1.068
Anxiety	0.267	0.386	0.715 to 2.384
Depression*	0.523	0.099	0.906 to 3.140
Helplessness*	0.04	0.175	0.982 to 1.102
Acceptance	−0.021	0.487	0.923 to 1.039
Disease benefits	0	0.988	0.948 to 1.056
Global health*	−0.009	0.167	0.978 to 1.004
Physical function	−0.006	0.274	0.983 to 1.005
Role function*	−0.007	0.106	0.985 to 1.001
Emotional function*	−0.01	0.114	0.978 to 1.002
Cognitive function*	−0.014	0.028	0.975 to 0.999
Social function*	−0.011	0.027	0.98 to 0.999
Fatigue*	0.011	0.032	1.001 to 1.022
Nausea	0	0.974	0.985 to 1.015
Pain	−0.001	0.819	0.99 to 1.008
Dyspnea	0.006	0.196	0.997 to 1.015
Insomnia	0.004	0.287	0.996 to 1.012
Loss of appetite	−0.001	0.802	0.989 to 1.008
Constipation	−0.002	0.664	0.987 to 1.008
Diarrhea	0.011	0.067	0.99 to 1.024
Financial problems	0.006	0.251	0.996 to 1.015
Necessity	−0.04	0.868	0.946 to 1.048
Concerns	0.025	0.362	0.971 to 1.082

*Statistically significant p < 0.20

### Multivariable analysis

We included the significant variables in univariable analyses in multivariables analysis. Using the backward stepping method, the variables - lower age (*p* = 0.003), fatigue (*p* = 0.013) and higher education level (*p* = 0.031) remained significant predictors for non-adherence. We checked for interactions between these three variables, but no significant interaction was found between any of the variables. The multivariable analysis revealed an area under the curve of 0.66 (95% confidence interval: 0.59–0.73) Table 4 shows the final multiple regression model to predict adherence.

**Table 4:** Multivariable analysis

Variable	*B*	*P* value	95% CI
Age*	−0.031	0.003	0.95 to 0.99
Fatigue*	0.014	0.013	1.00 to 1.03
Education level*	0.378	0.031	1.04 to 2.06
Diarrhea	0.009	0.169	1 to 1.02
Experiencing social support	0.786	0.2	0.66 to 7.30
Depression	0.396	0.296	0.71 to 3.12
Living alone	−0.354	0.327	0.35 to 1.43
Bisphosphonates	0.27	0.446	0.66 to 2.62
Working	0.225	0.526	0.63 to 2.51
Helplessness	0.023	0.603	0.94 to 1.11
Cognitive function	−0.004	0.61	0.98 to 1.01
Role function	0.003	0.678	0.99 to 1.02
Dyspnea	0.003	0.651	0.99 to 1.01
Global health	−0.005	0.671	0.97 to 1.02
Emotional function	0.001	0.935	0.98 to 1.02

AUC = 0.66

*Statistically significant *p* < 0.10

## Discussion

This study explored the prevalence of medication non-adherence and identified associated factors for non-adherence in hematological-oncological patients. In our study population, the prevalence of non-adherence was 50% [30]. This is comparable to other studies [5–7]. These results show us that it is necessary to take action to tackle medication non-adherence.

According to our prediction model, lower age is the most important risk factor for non-adherence. Also, fatigue and higher education level are strong predictors. Evidence from other studies on adherence in chronic patient populations showed that younger age is associated with lower adherence as well [13, 31–35].

Higher education was also found to be a predictor of medication non-adherence in other studies. [35, 36]

Dobbels et al. suggest that this may be due either to busier lifestyles or to the fact that higher educated patients are more ‘decisive’ non-adherers. According to a study amongst renal transplant patients decisive non-adherers often prefer to make independent decisions regarding their disease and treatment [31].

Also, fatigue was correlated to medication non-adherence in our study. This was measured as part of the quality of life questionnaire EORTC QLQ-C30. In a study in CML patients [37] fatigue was reported to have a negative influence on quality of life. A reduced quality of life may be a reason for poor adherence [11].

In our study, we used the MARS-5 questionnaire. It has no cut-off value to define adherence. We defined non-adherence as “a deviation from the prescribed medication regimen sufficient to adversely influence the regimen’s intended effect” [1]. In our opinion, a patient was considered non-adherent when he scored less than the maximum score of the MARS-5. This definition is strict, we did not allow patients to even forget their medication once and therefor stated that patients who did not score 25 on the MARS-5 are non-adherent. We chose this definition because of the seriousness of the diseases, complications or side effects patients are treated for. The MARS-5 is a validated questionnaire measuring non-adherence. However the MARS-5 is not validated in hematological patients, it has been used in other studies on non-adherence in hematological patients [38, 39].

### Limitations

Even though the response rate is satisfactory, it is possible that respondents with a more positive attitude returned the questionnaire; this might have influenced the results positively. Secondly, the data were gathered from self-reports. Although questionnaires were anonymous, respondents’ answers may not correspond with their actual behavior. Another limitation of this study is that we studied non-adherence at one university hospital only, which limits the extrapolation of our results. Thereby, this was a cross sectional study this study was cross-sectional therefore does not account for variations in patient responses over time and different scenarios. In the questionnaire we failed to explicitly mention that PRN medication should not be taken into account by filling in the MARS-5. Patients who would only use PRN medication were filtered out by checking their medical files. Finally, due to the high number of statistical tests being carried out in this research, statistical significance in the results may have reached by chance (type 1 error).

### Clinical implications

Half of our study population reported non-adherence to their prescribed medication. On the basis of these results, we started a questionnaire based screening program at admission to the clinical ward. The questionnaire will be used for further research on non-adherence, it includes factors associated to non-adherence as measured in this study (age, level of education and fatigue), factors of non-adherence according to the WHO (2003) [5] (factors of the health system and the treatment team, socio-economic factors, health-related factors, treatment-related factors and patient related factors) and the MARS-5 questionnaire. Next we review the questionnaires and specifically counsel patients who comply with the associated factors found in this study and patients who are non-adherent. Reasons of non-adherence should be investigated. Then goals can be set to prevent patients for being non-adherent during the treatment for their hematological malignancy.

Furthermore, this study gave insight into medication non-adherence and alerted doctors and nurses to address this subject with patients. Educating patients before and during therapy is of major importance for successful treatment [40]. Adherence rates should be estimated and this should be reported in the patient’s medical file to discuss adherence and to follow up on it.

Additionally, tools to improve adherence are available, but more research must be done to find out which ones are effective in patients with hematological malignancies.

## Conclusions

This cross-sectional study shows that the prevalence of non-adherence is high in hematological-oncological adult outpatients (50%) and that lower age of patients, fatigue and higher education level are associated factors. Although this study only provides a single baseline measurement, we feel that new strategies to address non-adherence are urgently needed in our patient population. Improvement of information supplied to patients at risk and adequate monitoring may be part of these strategies, but further research on this topic needs to be performed.

## Abbreviations

ALL: Acute lymphoid leukemia
BMQ: Beliefs about medication questionnaire
CML: Chronic myeloid leukemia
EORTC QLQ-C30: European Organization for Research and Treatment of Cancer, Quality of Life Questionnaire-C 30 version 3.0
HADS: Hospital anxiety and depression subscale
ICQ: Illness cognitions questionnaire
MARS-5: Medication adherence rating scale 5 item version
WHO: World Health Organization
